# Interactions Between Diabetes Mellitus and Osteoarthritis: From Animal Studies to Clinical Data

**DOI:** 10.1002/jbm4.10626

**Published:** 2022-04-23

**Authors:** Naiomy D. Rios‐Arce, Nicholas R. Hum, Gabriela G. Loots

**Affiliations:** ^1^ Physical and Life Sciences Directorate, Lawrence Livermore National Laboratories Livermore CA USA; ^2^ Molecular and Cell Biology, School of Natural Sciences University of California Merced Merced CA USA

**Keywords:** CARTILAGE, DIABETES, TYPE 1 DIABETES, OSTEOARTHRITIS, POSTTRAUMATIC OSTEOARTHRITIS, TYPE 2 DIABETES

## Abstract

Diabetes mellitus (DM) and osteoarthritis (OA) are commonly known metabolic diseases that affect a large segment of the world population. These two conditions share several risk factors such as obesity and aging; however, there is still no consensus regarding the direct role of DM on OA development and progression. Interestingly, both animal and human studies have yielded conflicting results, with some showing a significant role for DM in promoting OA, while others found no significant interactions between these conditions. In this review, we will discuss preclinical and clinical data that assessed the interaction between DM and OA. We will also discuss possible mechanisms associated with the effect of high glucose on the articular cartilage and chondrocytes. An emerging theme dominates the breath of published work in this area: most of the studies discussed in this review do not take into consideration the role of other factors such as the type of diabetes, age, biological sex, type of animal model, body mass index, and the use of pain medications when analyzing and interpreting data. Therefore, future studies should be more rigorous when designing experiments looking at DM and its effects on OA and should carefully account for these confounding factors, so that better approaches can be developed for monitoring and treating patients at risk of OA and DM. © 2022 The Authors. *JBMR Plus* published by Wiley Periodicals LLC on behalf of American Society for Bone and Mineral Research.

## Introduction

Diabetes mellitus (DM) and osteoarthritis (OA) are chronic conditions that together affect about 1 billion people worldwide^(^ ^1^ ^)^ (Centers for Disease Control and Prevention [CDC], The Facts, Stats, and Impacts of Diabetes; https://www.cdc.gov/diabetes/library/features/diabetes-stat-report.html). Diabetes is a metabolic disease that results in elevated blood glucose, whereas OA is a chronic joint condition that results in pain, stiffness, and diminished mobility of the affected joints. Both conditions can have a significant impact on the quality of life of patients. Furthermore, these two conditions share several risk factors such as obesity and aging.^(^ ^2^, ^3^, ^4^, ^5^, ^6^ ^)^ Early studies looking at the interaction between DM and OA found a higher prevalence of knee OA in DM patients; however, this effect was attributed to an increase in body weight, an effect seen more often in type 2 diabetes (T2D) patients.^(^ ^7^ ^)^ Interestingly, other studies showed the development of hand OA in DM patients, opening the possibility that the link between DM and OA is not solely based on weight bearing and mechanical factors, and that indeed DM might play a role in OA development. In this review we will discuss animal and clinical studies that assessed the association between DM and OA. We will also describe some of the mechanisms associated with the interplay between DM and OA.

## DM

The World Health Organization (WHO) estimates that more than 422 million people worldwide have DM and around 1.5 million deaths are directly attributed to this condition, annually (WHO, Diabetes; https://www.who.int/news-room/fact-sheets/detail/diabetes). DM refers to a group of metabolic diseases in which the human body is unable to regulate blood glucose levels resulting in hyperglycemia. There are three types of DM: type 1 diabetes (T1D), T2D, and gestational diabetes. T1D is commonly diagnosed in children and young adults; it is greatly influenced by genetic factors and affects nearly 1.6 million Americans. During T1D the pancreas produces little or no insulin resulting in dysregulated blood glucose levels.^(^ ^8^ ^)^ T2D, on the other hand, is the most common type of DM and according to the CDC, T2D accounts for up to ~95% of DM diagnoses in the United States. T2D mostly manifests in adults, and it occurs when the body does not produce enough insulin, or it becomes resistant to it. T2D is associated with obesity, and it can be managed by changes in diet, exercise, and weight control.^(^ ^8^, ^9^ ^)^ Gestational diabetes, the least common type of DM, is diagnosed in expectant mothers during pregnancy and usually goes away after delivery.^(^ ^10^, ^11^ ^)^ The effects of DM on patients' health have been well studied. DM has widely been associated with kidney (nephropathy), nerve (neuropathy), and eye (retinopathy) damage. In addition, DM has also been shown to influence bone mineral density by affecting the bone remodeling process and contributing to a low bone mass phenotype.^(^ ^12^, ^13^, ^14^ ^)^ In recent years DM has also been found to influence OA development.^(^ ^7^, ^15^, ^16^, ^17^, ^18^, ^19^, ^20^, ^21^ ^)^

## OA

**Fig. 1:**
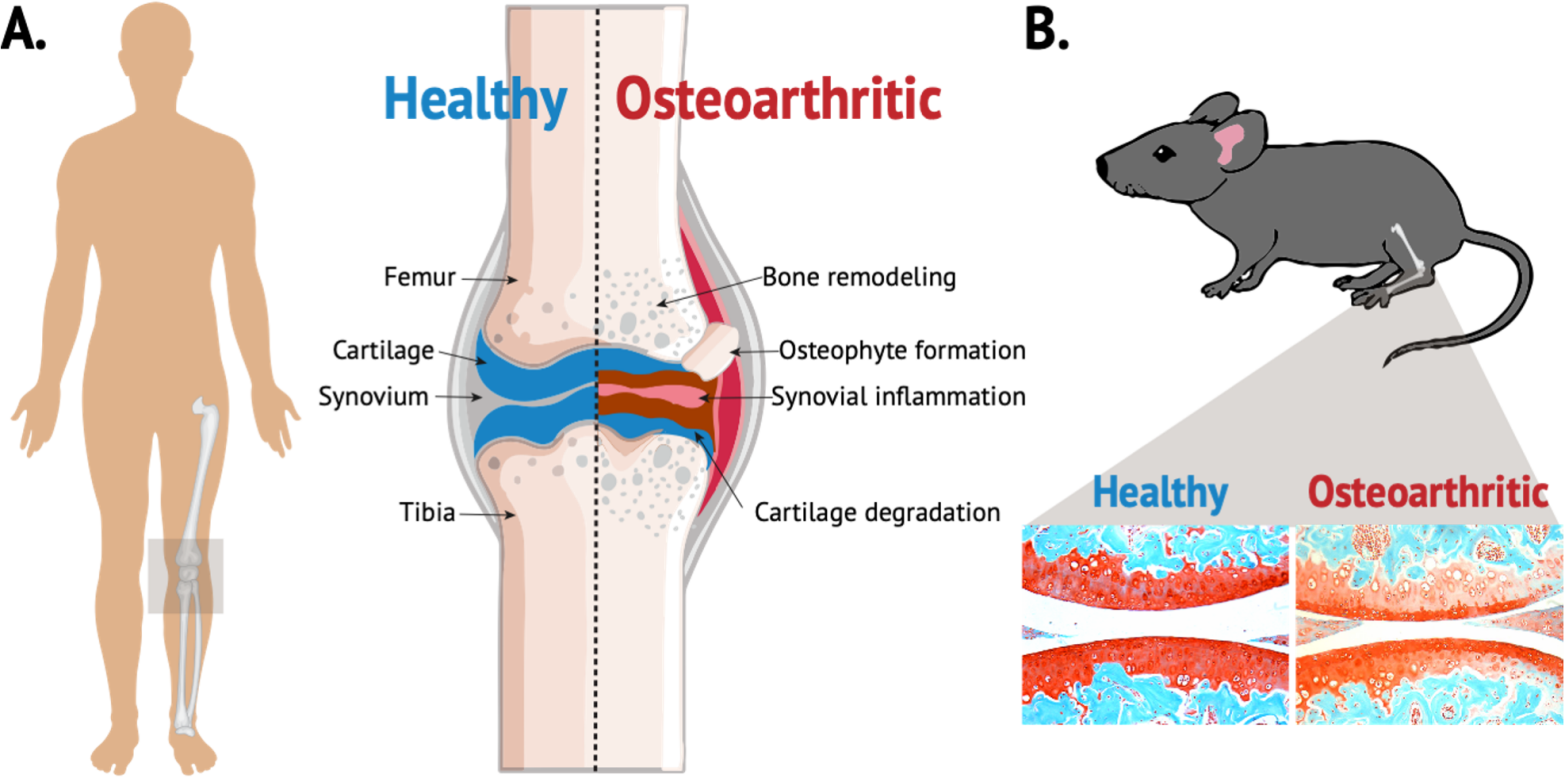
Schematic representation of an osteoarthritic knee joint. (*A*) Illustration of main characteristics of healthy versus osteoarthritic knee joint. (*B*) Histological representation of osteoarthritis in a mouse model using Safranin O (red: cartilage) and Fast Green (green: surrounding tissue) staining.

Osteoarthritis (OA) is a painful degenerative joint disorder that affects around 654 million individuals aged ≥40 years, worldwide.^(^ ^1^ ^)^ In the United States, OA is the most common type of arthritis, affecting approximately 32.5 million adults.^(^ ^1^ ^)^ OA is considered to be one of the most expensive conditions to treat when joint replacement surgery is required, and it is estimated that around 1 million knee and hip replacement surgeries are performed each year.^(^ ^22^ ^)^ OA is also becoming the third most rapidly rising condition associated with disability.^(^ ^1^ ^)^ OA is characterized primarily by articular cartilage loss, synovial inflammation, and ectopic bone formation in the affected joint (Fig. 1*A*). This chronic and complex joint disease can affect any joint; however, the most common joints affected are the hands, lower back, neck, and weight‐bearing joints such as knees, hips, and feet.^(^ ^23^ ^)^ OA can be broadly classified in two different forms, primary and secondary. Primary or idiopathic OA is considered “wear and tear” and is mostly related to aging; therefore, it starts showing up in people between the ages of 55 and 60 years. Several studies have also indicated a hereditary component to primary OA,^(^ ^24^ ^)^ and these patients may develop symptoms significantly earlier.

Secondary OA involves a specific trigger that intensifies cartilage breakdown. These triggers can include injury, overuse, obesity, genetics, inactivity, inflammation, and/or other diseases.^(^ ^25^ ^)^ Individuals who sustain a joint injury are known to be at substantially increased risk of developing OA compared with uninjured persons; OA that develops after a joint injury is referred to as posttraumatic OA (PTOA). PTOA accounts for ~12% of all diagnosed OA cases. Among youth and athletes, knees and ankles are the most injured joints; sprains and strains to these joints make up about 11% of all musculoskeletal injuries treated in the United States. Among knee injuries, 23% involve the meniscus and 25% involve the anterior cruciate ligament (ACL). In general, ACL tears are frequently accompanied by additional damage to other parts of the joint, including articular cartilage and subchondral bone, collateral ligaments, and menisci. It is estimated that concurrent meniscal damage occurs in up to 75% patients who sustained an ACL injury. Prospective studies have highlighted that as many as 59% of patients who experienced an ACL or meniscal tear would go on to develop OA 10 to 20 years after the injury, independent of whether or not surgical reconstruction occurred. Although primary and secondary OA are caused by different factors, the resulting pathology is the same: cartilage degeneration, joint pain, and stiffness. OA also affects bone remodeling and can lead to bone loss or ectopic ossification.^(^ ^26^, ^27^ ^)^ OA is an irreversible disease and no pharmacological interventions exist that can help patients repair and rebuild lost cartilage. When symptoms cannot be controlled, and mobility is significantly impaired, total joint replacement surgery remains the only corrective option for OA patients.

## Animal Studies

### Idiopathic OA

Animal studies have been widely used to examine the effects of DM on the onset and progression of OA (Fig. 1*B*); however, similar to human studies, there has been no consensus reached regarding the role of DM on OA development. The discrepancy in animal studies can be in part explained by several factors including, but not limited to: (i) choice of animal model (mouse versus rat), (ii) differences in age of animals used (young versus old), (iii) different assessments of OA development, (iv) different types of diabetes model (T1D versus T2D), and (v) differences in types of injury models used. Here we summarize the results from these published studies to highlight differences as well as commonalities in results and conclusions.

In a study using Lewis rats, 6 to 12 months of age, mixed biological sex, it was found that the rats with T2D had a significantly higher mean score of OA in the medial and lateral femur and medial tibia than the nondiabetic controls, suggesting that T2D can be a factor in the onset and progression of OA.^(^ ^15^ ^)^ However, in this study increased body mass could potentially be a confounding factor because the T2D rats were significantly heavier (more than twofold) than nondiabetic rats. OA phenotypes of increased severity have been significantly correlated with animals of greater mass.^(^ ^15^ ^)^ Another study, using young Wistar rats treated with streptozotocin (STZ) for 70 days, showed less proteoglycan content and collagen II expression in the joints of T1D rats than the nondiabetic controls, suggesting that T1D can promote OA development in this rat model.^(^ ^17^ ^)^ One advantage of this study was that the T1D rats were lean; therefore, body mass did not play a role in the phenotype observed.^(^ ^17^ ^)^ Similarly, after 8 weeks of diabetes induction via STZ treatment, Sprague‐Dawley rats displayed significant cartilage damage in their knee joints compared to the control nondiabetic group.^(^ ^28^ ^)^ STZ treated mice also showed enhanced cartilage damage and lower expression of collagen II when compared to the control mice.^(^ ^18^, ^29^, ^30^ ^)^

Consistent with the T1D rat models, work from our group has recently shown a significant loss of proteoglycan content in an STZ‐induced T1D C57BL/6 mouse model at 16 weeks of age.^(^ ^31^ ^)^ C57BL/6 male mice on a high fat (HF) diet (as a model of T2D) had larger osteophytes and displayed more synovial hyperplasia than control mice on regular chow.^(^ ^32^ ^)^ Comprehensively, all of these studies support a role for DM in OA development with the exception of one study involving mice in which no association was found between DM and OA.^(^ ^16^ ^)^ In that study, by Ribeiro and colleagues,^(^ ^16^ ^)^ a genetic model (*db/db*) of diabetic mice was examined histologically to determine OA status. These mice were obese, hyperglycemic, hyperinsulinemic, and insulin resistant. However, contrary to HF‐induced T2D studies, *db/db* joints showed no significant difference in the knee cartilage relative to wild‐type control mice, suggesting that different mechanisms of action lead to OA development in these two obese, T2D mouse models.^(^ ^16^ ^)^

### Trauma‐induced OA

Because injury is a well‐known risk factor for OA,^(^ ^25^, ^33^ ^)^ several studies have also assessed the role of DM on trauma‐induced OA. Ten weeks after surgical induction of OA by transection of medial meniscotibial and medial collateral ligament (DMM), obese, T2D *db/db* mice showed increase in cartilage damage, inflammation, and loss of proteoglycan content when compared to control lean injured mice.^(^ ^16^ ^)^ C57BL/6 mice on a HF diet also accelerated the progression of OA after meniscal/ligamentous injury (MLI). These animals had increased fibrillation, clefting, and lost 25% to 50% of their articular cartilage 2 months after MLI.^(^ ^34^ ^)^ In contrast to these findings, work from our group has determined a significantly slower progression toward an OA phenotype after knee injury in STZ‐treated C57BL/6 mice, a model of T1D. Using a noninvasive tibial compression injury model,^(^ ^35^, ^36^, ^37^ ^)^ T1D mice developed significantly milder PTOA phenotypes following joint injury than the nondiabetic injured control mice. This protection seemed to be driven by an increase in chondrocytes in the articular cartilage which may be due to resistance to apoptosis, postinjury, in T1D joints.^(^ ^31^ ^)^

## Clinical Data

The role of diabetes on OA progression has also been studied in patients; here, similar to animal models, results have yielded inconsistent and conflicting conclusions. Two systematic review studies found a higher risk of OA development in DM patients when compared to a non‐DM control group.^(^ ^7^, ^38^ ^)^ Louati and colleagues^(^ ^7^ ^)^ examined 49 studies (*n* = 1,192,518) that included both T1D and T2D patients in their analysis. Overall, they found an elevated risk of OA in the DM population when compared to the non‐DM population. In addition, seven studies identified DM as an independent risk factor specifically for knee and hand OA when they adjusted for body mass index (BMI). However, five of the 49 studies did not show an association between DM and OA.^(^ ^7^ ^)^ The second systematic review included 10 articles on the topic of T2D and OA. The pooled population size in their meta‐regression analysis included 16,742 patients and they also found a significant association between T2D and the development or presence of OA. Similar to Louati and colleagues,^(^ ^7^ ^)^ seven of these studies (*n* = 7156), that did control for weight or BMI, showed an association between T2D and OA.^(^ ^38^ ^)^

Several other studies have also supported the idea of DM as an independent risk factor for OA. One study examining metabolic risk factors and their role in knee OA showed a significant association between impaired glucose tolerance (IGT) and knee OA after adjusting for age and biological sex, but not for BMI.^(^ ^39^ ^)^ A longitudinal study by Schett and colleagues^(^ ^40^ ^)^ showed that T2D comprised a twofold risk of severe OA resulting in arthroplasty. Results were consistent when the data was normalized to age and BMI.^(^ ^40^ ^)^ A cross‐sectional study performed in 202 subjects from Puerto Rico showed that the prevalence of OA in patients with DM was 49%, whereas only 26.5% of individuals without DM had OA; in this study females with DM were more likely to have hand or knee OA than males. After adjusting for age, biological sex, education level, obesity, exercise, and osteoporosis, DM patients had 2.18‐fold increased risk of hand or knee OA compared to nondiabetic subjects.^(^ ^41^ ^)^ A 3‐year follow‐up study, which included 559 patients >50 years, showed that T2D male patients developed a narrower joint space than females, suggesting a gender effect for DM on OA progression.^(^ ^20^ ^)^ Interestingly, the effects of DM on OA also seemed to be age‐dependent, as shown in the Rotterdam Study.^(^ ^42^ ^)^ In that study, an association between DM and hand OA was only seen in people aged 55 to 62 years, but not in the total population or in other age groups after adjusting for age, biological sex, smoking, and BMI.^(^ ^42^ ^)^ In a slightly different study looking at DM and knee pain in patients with OA it was determined that patients with DM were about 2.5 times more likely to have knee pain than subjects with knee OA without DM, after adjusting for several covariates, such as age, biological sex, BMI, and the use of pain medications.^(^ ^43^ ^)^ Another, smaller study, that included only 23 diabetic and 47 nondiabetic patients with OA undergoing total knee arthroplasty, also showed that DM patients had on average a higher knee injury and OA outcome pain score than nondiabetic patients.^(^ ^44^ ^)^ Less studied, DM has also been associated with pain in hand OA.^(^ ^21^ ^)^

In contrast to the aforementioned studies, a more recent meta‐analysis and several other studies have shown no association or an inverse association between DM and OA. A study by Horn and colleagues,^(^ ^45^ ^)^ in a population of 25 females with DM, showed that osteophytes were less common in the DM patients than in controls. Sixteen percent and 2% of the DM and non‐DM patients, respectively, showed no evidence of osteophyte formation.^(^ ^45^ ^)^ Similarly, The Korea National Health and Nutrition Examination Survey (KNHANESV‐1) nationwide survey showed no association between insulin resistance and risk of OA when BMI was included.^(^ ^46^ ^)^ Interesting, the Singapore Chinese Health Study showed a significant inverse association between DM and risk of total knee replacement.^(^ ^47^ ^)^ One important detail of this study is that the BMI between DM and non‐DM subjects was not significantly different, completely assessing the effect of DM on total knee replacement due to DM independently of obesity or body weight.^(^ ^47^ ^)^ However, this inverse association might be explained by the fact that people with DM are less likely to go to surgery due to possible complications; therefore, more studies are needed to completely understand the relationship to OA. Similarly, a systematic review, which included 31 independent studies (*n* = 295,100), did not support the idea that DM is an independent risk factor for OA and suggested that an increase in body mass is the main driver in promoting OA. Of these 31 studies, 16 showed a positive association and 15 reported null or no association between DM and OA. In addition, only 68.8% of the studies that showed an association between DM and OA adjusted for BMI, whereas 93.3% of the studies that showed no effect adjusted for this variable. Furthermore, the studies that showed no association had larger samples sizes than the ones with positive associations.^(^ ^48^ ^)^ The Maastricht Study, a self‐reported knee pain study, showed no association between T2D and knee OA after adjusting for BMI.^(^ ^19^ ^)^ In terms of hand OA, Frey and colleagues^(^ ^49^ ^)^ and Garessus and colleagues^(^ ^50^ ^)^ observed no statistically significant association between T2D and hand OA.

The differences in the results obtained across all these studies can in part be attributed to the contribution of other OA common risk factors, such as obesity and aging; these variables were taken into consideration in some of the studies, but not in most of them. For example, one of the studies that suggested that DM can have a negative effect on OA did not account for the contribution of body mass changes.^(^ ^51^ ^)^ In addition, only two studies described their population in terms of the type of DM the patients suffered from (T1D versus T2D); however, T2D patients were most common to both studies.^(^ ^7^ ^)^

These clinical findings have not been able to conclusively establish the exact role DM plays in OA, specifically in humans. Clinical studies can provide key findings regarding the interaction between DM and OA; however, they also present several challenges. For example, DM patients often receive treatments to control their blood glucose levels or for other conditions exacerbated by DM. These treatments may have cofounding effects on bone and cartilage—something that is not often described in clinical studies. In addition, DM is not diagnosed right away and the CDC estimates that around 96 million people aged ≥18 years have prediabetes (CDC, National Diabetes Statistics Report; https://www.cdc.gov/diabetes/data/statistics-report/index.html); therefore, it is challenging to determine if the duration of DM in a patient contributes to OA development or progression. Furthermore, to our knowledge, no studies to date have examined the role of gestational diabetes on OA development. In summary, more studies are needed that will take into consideration all of the other factors that can affect and contribute to DM and OA to better understand their relationship.

## Mechanisms of Action Linking Diabetes and OA Development

The molecular mechanisms underlying the connection between DM and OA have been minimally explored; however, several studies have proposed a role of systemic inflammation, mechanical stress, accumulation of advanced glycation end products (AGEs), and oxidative stress as part of this mechanism. The articular cartilage, a nonvascularized and non‐innervated tissue, is composed of a dense extracellular matrix (ECM), mostly made of water, collagen, proteoglycans, and specialized cells known as chondrocytes.^(^ ^52^ ^)^ The role of this tissue is to reduce friction between the bones and to facilitate the transmission of loads to the subchondral bone.^(^ ^52^ ^)^ Studies have shown that chondrocytes are highly glycolytic cells that can sense and adjust to glucose variations in their environment. Chondrocytes respond to glucose changes through the expression of the glucose transporters 1, 3, and 9 (GLUT‐1, GLUT‐3, and GLUT‐9).^(^ ^53^ ^)^ During low extracellular glucose concentrations (5–10mM), healthy chondrocytes increase GLUT‐1 transporter expression, whereas during high extracellular glucose concentrations (25–75mM) they decrease their levels. In a study using human chondrocytes Rosa and colleagues^(^ ^54^ ^)^ determined that chondrocytes harvested from healthy individuals are effective in adjusting themselves to variations in glucose levels, whereas chondrocytes isolated from OA patients, exposed to high glucose levels, were defective in regulating GLUT‐1 expression, resulting in the accumulating of glucose and increased reactive oxygen species (ROS) production. In support of this hypothesis, a different study by Hosseinzadeh and colleagues^(^ ^55^ ^)^ showed that high glucose concentrations can induce oxidative stress and mitochondrial apoptosis in C28I2 human chondrocytes. Furthermore, hyperglycemia‐induced catabolic and inflammatory responses in chondrocytes can be mediated via toll‐like receptor 4 (TRL4) through exacerbating the activation of the transcription factor nuclear factor κB (NF‐κB),^(^ ^56^ ^)^ a phenomenon also supported by administering lipopolysaccharide (LPS) prior to joint injury.^(^ ^57^ ^)^ In addition, high glucose concentrations (25–75mM) can also result in the reduction of chondrogenic differentiation and an increase in matrix metalloproteinases (MMPs).^(^ ^58^, ^59^ ^)^ Interesting, one study showed that low glucose concentrations upregulate aggrecan expression and stimulate chondrogenesis in mouse chondrocytes, suggesting that low glucose concentrations can prevent OA, whereas high concentrations of glucose can promote OA development.^(^ ^60^ ^)^

One of the most well‐studied mechanisms associated with OA development is inflammation. It has been demonstrated that during OA a biological trigger and/or mechanical stress can lead to the infiltration of inflammatory cells (eg, lymphocytes and macrophages) that enhance the secretion of pro‐inflammatory cytokines such as tumor necrosis factor alpha (TNFα), interleukin‐1 beta (IL‐1β), IL‐17, ROS, and AGEs. This local inflammation further leads to the production of proteolytic enzymes such as MMPs and aggrecanases, which induce cartilage degradation.^(^ ^61^, ^62^ ^)^ An increase in chondrocytes apoptosis has also been suggested to play a role in OA development.^(^ ^63^ ^)^ MMPs have been associated with OA development because they can promote cartilage degradation, and studies in humans have found elevated expression levels of some MMPs in the serum of OA patients. High levels of MMPs 1, 7, 8, 9, 10, and 12 have been previously determined in the serum and synovial fluid of DM patients with OA relative to controls^(^ ^64^ ^)^; however, how much MMPs contribute to OA development and which factors/conditions contribute to their elevated expression in OA patients is not completely understood. In animal models of PTOA, several MMPs have been shown by immunohistochemistry and mRNA to be elevated in the articular cartilage, post injury.^(^ ^35^, ^65^ ^)^

Elevated inflammation and inflammatory associated molecules seem to also play a role in DM‐induced OA. Higher synovitis and elevated levels of IL‐6 in human DM knee joints have been determined. Interesting, the level of synovitis in DM patients was also associated with knee pain.^(^ ^44^ ^)^ Stimulation of human knee explants from T2D patients with IL‐1β, as an inflammatory response, enhanced IL‐6 and prostaglandin E_2_ (PGE2) expression.^(^ ^66^ ^)^ IL‐1β stimulation of cultured chondrocytes under high glucose concentration also resulted in greater production of ROS and nitrous oxide.^(^ ^66^ ^)^ One study in T1D Sprague‐Dawley rats found a twofold to fivefold increase in TNFα and IL‐6 expression in the serum of T1D rats when compared to control rats.^(^ ^28^ ^)^ Another animal study showed the involvement of the peroxisome proliferator‐activated receptor gamma (PPARγ) in inflammation and DM‐induced OA. In this study it was determined that high glucose concentration in human chondrocytes can downregulate PPARγ and collagen II expression and stimulate the expression of cyclooxygenase‐2 (COX‐2) and the production of PGE2, MMP13, and IL‐6. Furthermore, they showed that Pioglitazone, an agonist of PPARγ, can partially reverse cartilage erosion in DM mice and can inhibit the production of PGE2, MMP13, and IL‐6. These results suggest that regulation of PPARγ can prevent inflammation and can therefore prevent DM‐induced OA.^(^ ^18^ ^)^

Human chondrocytes and mouse studies from DM groups have shown reduced autophagy, suggesting that defective autophagy might be another mechanism that can explain cartilage degradation in DM conditions.^(^ ^16^, ^67^ ^)^ Autophagy is an important process in cartilage maintenance, and treatment with Rapamycin, a pharmacological activator of autophagy, prevented cartilage degradation, proteoglycan loss, inflammation, and decreased MMP13 expression in experimentally induced *db/db* OA mice.^(^ ^16^ ^)^ These results demonstrate that autophagy regulation in experimentally induced OA is an important factor, and that autophagy activation can prevent OA development in obese T2D mice.

Treatment with adipose‐derived stem cells (ADSCs) has also yielded a beneficial effect in preventing DM‐induced OA. STZ‐treated mice showed cartilage degradation with increases in MMP13. Intraarticular administration of ADSCs for 4 weeks prevented this effect; ADSC‐treated DM‐OA group joints had a smoother articular surface and higher chondrocyte numbers when compared to a control OA group. These results suggest that ADSCs treatment can prevent inflammation and cartilage degeneration in T1D mice.^(^ ^29^ ^)^

## Conclusion

Both animal and human studies to date have yielded conflicting and inconsistent results linking DM with OA initiation and progression. Although across all the studies discussed in this review there is a general consensus that DM is an independent risk factor for OA and joint pain, future studies will need to be more rigorous and take into account several covariates, such as age, biological sex, BMI, the use of (pain) medications, and the type of DM. In addition, there is a lack of studies looking at the role of gestational diabetes effects on OA.

## Author Contributions

**Naiomy D Rios‐Arce:** Data curation; writing – original draft; writing – review and editing. **Nicholas R Hum:** Visualization. **Gabriela G Loots:** Conceptualization; data curation; writing – original draft; writing – review and editing.

## Conflict of Interest

The authors declare no conflict of interest.

### Peer Review

The peer review history for this article is available at https://publons.com/publon/10.1002/jbm4.10626.
